# A Proprioceptive Soft Robot Module Based on Supercoiled Polymer Artificial Muscle Strings

**DOI:** 10.3390/polym14112265

**Published:** 2022-06-01

**Authors:** Yang Yang, Honghui Zhu, Jia Liu, Haojian Lu, Yi Ren, Michael Yu Wang

**Affiliations:** 1 School of Automation, Nanjing University of Information Science and Technology, Nanjing 210044, China; 20201249181@nuist.edu.cn; 2 State Key Laboratory of Industrial Control and Technology, Institute of Cyber-Systems and Control, Zhejiang University, Hangzhou 310027, China; luhaojian@zju.edu.cn; 3 Robotics X Lab, Tencent, Shenzhen 518000, China; evanyren@tencent.com; 4 Department of Mechanical and Aerospace Engineering, The Hong Kong University of Science and Technology, Hong Kong, China; mywang@ust.hk; 5 Department of Electronic and Computer Engineering, The Hong Kong University of Science and Technology, Hong Kong, China

**Keywords:** soft robot module, SCPAM strings, proprioceptive sensing, actuation–sensing integration, crawling, gripping

## Abstract

In this paper, a multi-functional soft robot module that can be used to constitute a variety of soft robots is proposed. The body of the soft robot module made of rubber is in the shape of a long strip, with cylindrical chambers at both the top end and bottom end of the module for the function of actuators and sensors. The soft robot module is driven by supercoiled polymer artificial muscle (SCPAM) strings, which are made from conductive nylon sewing threads. Artificial muscle strings are embedded in the chambers of the module to control its deformation. In addition, SCPAM strings are also used for the robot module’s sensing based on the linear relationship between the string’s length and their resistance. The bending deformation of the robot is measured by the continuous change of the sensor’s resistance during the deformation of the module. Prototypes of an inchworm-like crawling robot and a soft robotic gripper are made, whose crawling ability and grasping ability are tested, respectively. We envision that the proposed proprioceptive soft robot module could potentially be used in other robotic applications, such as continuum robotic arm or underwater robot.

## 1. Introduction

The majority of the body of soft animals in nature consist of soft tissues, whose mechanical properties allow animals to store elastic energy, cushion impacts, and endow them with great environmental adaptability [1]. Inspired by such animals, researchers have designed and developed a variety of soft robots. These robots tend to be more flexible, conformable, and adaptable to unstructured environments. These features enable them to interact with human beings safely.

Various soft robots available in different shapes and functions have been developed to achieve bionic motion. The major actuation methods of these soft robots include: shape memory alloy (SMA) [2,3,4], pneumatic actuator [5,6,7], dielectric elastomer actuator (DEA) [8,9,10], ionic polymer–metal composite (IPMC) [11,12,13], motor & tendon [14,15,16], liquid crystal elastomer (LCE) [17,18,19], shape memory polymer (SMP) [20,21], etc. Modularity is a current trend in the field of soft robotics, which means that general adaptive soft robot modules are developed and assembled into soft robots with different functions according to actual needs.

Table 1 lists the soft robot modules built with common actuation methods, as well as their soft robotic applications [16,22,23,24,25,26,27,28,29,30], and each of these actuation methods has its own pros and cons. Among them, SMA is classified as a metallic material and has a nonnegligible hysteresis in the phase transformation process [31]. Pneumatic actuators are considered as soft and biocompatible, but they often require bulky accessories such as air compressors, which significantly limits the practical application in self-contained robotic systems. DEAs have a low Young’s modulus, high elastic energy density, and exhibit a strain of up to 200%. The fast-responding features of DEAs makes them particularly suitable for the fast driving of bionic robots [32,33]. However, several kilovolts are usually required to reach the full strain, and additional voltage amplifiers are needed to output high voltages. The overall performance of the DEAs is highly dependent on the elastomer stiffness, dielectric constant, and breakdown voltage [34]. The downsides of DEAs include leakage currents and the need for kilovolts of actuation voltage, which increases the risk of electrical breakdown. Currently, low-voltage stacked DEAs (LVSDEAs), which require less than 450-V operating voltages, have been reported and demonstrated to actuate insect robots [35]. IPMCs are ideal for biomimetic devices and require only a few volts to operate. However, they tend to have slow response times, low electromechanical coupling, and must be immersed in an electrolyte or encapsulated to operate in the air [36]. LCEs have good mechanical properties, great flexibility, and excellent anisotropic behaviors, but the processing technology for LCEs is still in the preliminary stage, which makes it difficult to control their deformation and motion accurately [37]. SMP actuators have advantages, including high strain recovery, biocompatibility, and biodegradability. However, they have some drawbacks, such as fatigue and long response time [31]. The motor & tendon driving approach is easy to control and responsive, but attached motors make the actuation system complicated, and the rigid parts added may affect the inherent compliance of the robot [38].

Coils formed by the constant twisting of polymer fibers or threads—namely, supercoiled polymer artificial muscle (SCPAM) strings—have been recently proposed as relatively novel artificial muscles and developed some applications in the field of soft robotics. Some specific applications include: crawling [39], grasping [40], swimming [41], etc. Some multifunctional soft robot modules driven by SCPAM strings have also been designed and fabricated. Tang et al. proposed a general robot module actuated by SCPAM strings that could be configured into a variety of robots. They fabricated a two-finger soft gripper to verify the versatility of the module [30]. In Tang et al.’s research, the sensing capability of SCPAM string is not discussed. Besides using SCPAM as the actuator of robots, some research results have already shown that it is feasible to use SCPAM as an internal strain sensor. The one-ply SCPAM string was embedded into the robot to estimate the externally applied load and length change of the robot based on the established model and measured resistance values during stretching or twisting [42]. Additionally, Zhao et al. integrated stretchable optomechanical thin film sensors into SCPAM strings to obtain SCPAM strings with sensing capability, and the SCPAM strings appeared as different colors for different strains under the impact of the current [43]. It has been reported that two-ply SCPAM can also be used as the strain sensor. In Bombara et al.’s study, they employed Preisach models and third-order polynomials to capture the relationship between SCPAM string resistance and length of the twisted string actuator (TSA), respectively [44,45]. Then, the length of TSAs can be estimated by using the resistance values as inputs. In this study, two-ply SCPAM strings are used as the actuator and sensor of the soft robot separately, which means only one of the above functions is available for a single two-ply SCPAM. Unless otherwise noted, SCPAM string below always refers to a two-ply SCPAM string.

**Figure 1:**
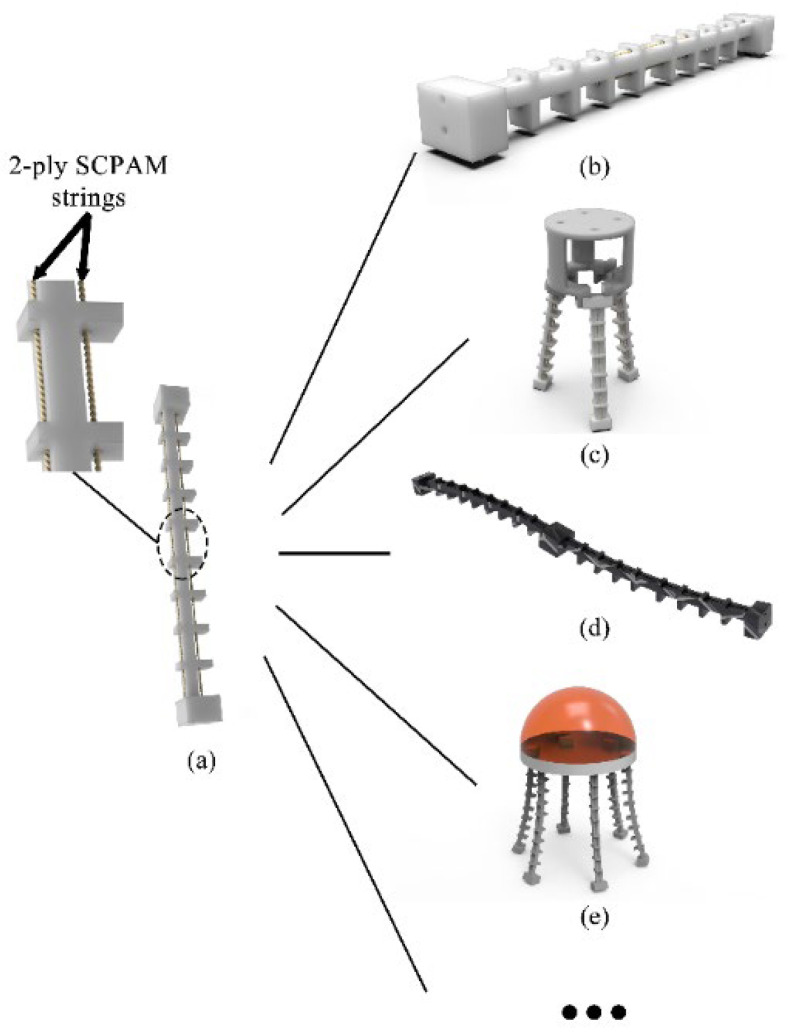
A single soft robot module and soft robots configured by the modules. (**a**) A single soft robot module, and chambers to configure the actuator and sensor. (**b**) An inchworm-inspired crawling robot. (**c**) A soft robotic gripper. (**d**) A snake-like manipulator. (**e**) A jellyfish-inspired robot with underwater swimming capability.

For the proprioceptive sensing of soft robots, traditional sensors such as strain gauges, encoders, and inertial measurement units (IMUs) cannot be applied directly and might impede the inherent compliance of soft robots. Recently, new perception solutions using self-conductive materials have provided a promising alternative for soft robots [46,47,48]. It has been reported that DEAs can rely on their own sensing signals to sense strain. The sensing signals can be divided into a force signal [49], capacitive signal [50], current signal [51], and a combination of voltage and current signal [52]. Similarly, different sensing signals based on SMAs can also sense strain [53,54]. In this research, we propose a soft robot module driven by SCPAM strings with a proprioceptive sensing capability. The module possesses bidirectional actuation capability and an integrated actuation–sensing function, which means that the robot module can bend in two opposite directions, and the internal soft strain sensor can be used to infer the bending deformation of the robot. As shown in Figure 1, we fabricate prototypes of a crawling robot and a robotic gripper using the soft robot module as a basic unit and envision other applications of soft robots based on the module, including a snake-like manipulator and a jellyfish-inspired robot with underwater swimming capability, etc. Even though lots of research have discussed the applications of SCPAM as the actuator or sensor separately, few of them have discussed the actuation–sensing integration of SCPAM strings. In this study, the actuation or sensing role of a SCPAM string can be adaptively altered based on the task requirement. Contributions of this research are:

(1) A novel proprioceptive soft robot module is proposed based on a 3D-printed soft body and SCPAM strings;
(2) Both the actuation and sensing properties of the SCPAM strings are utilized to realize the bending deformation, as well shape estimation, of the soft robot module;
(3) Based upon the soft robot module, a crawling robot and a robotic gripper are presented, and more soft robotic applications could be achieved.

The remainder of this article is organized as follows. Section 2 describes the design and fabrication of the soft robot module. In addition, the configurations of the crawling robot and the soft gripper are also elucidated. Section 3 tests the performances of the crawling robot and the robotic gripper, respectively, and demonstrates the actuation–sensing integration capability of the module. Finally, Section 4 summarizes the paper and gives an outlook towards future work.

## 2. Design and Fabrication

### 2.1. Design and Fabrication of the Soft Robot Module

**Figure 2:**
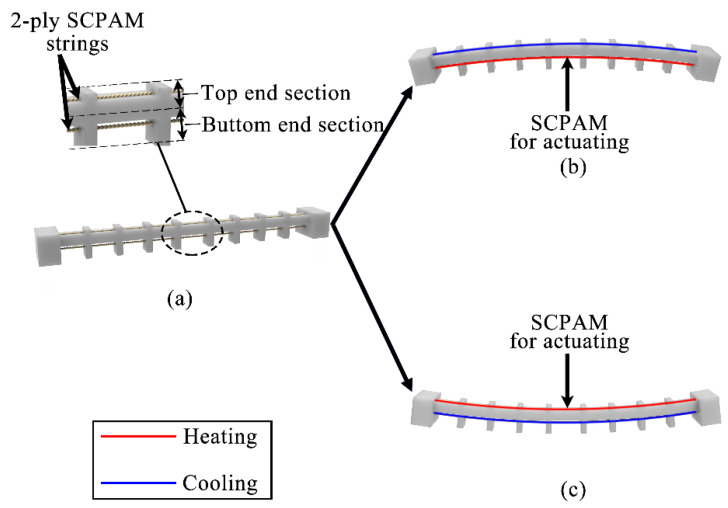
Definitions of forward bending and backward bending for the module. Red line means the power is on, and blue line means the power is off for the SCPAM strings. (**a**) The initial state of the soft robot module, as well as the definitions of the top and bottom end sections of the module. (**b**) Forward bending mode. (**c**) Backward bending mode.

The shape of the soft robot module is similar to a common soft manipulator whose length is 155 mm and maximum width is 18 mm. Two chambers are configured at the top end and bottom end of the robot module, respectively. Each of the chambers is capable of being configured with an actuator or a sensor, allowing the soft module to obtain bending deformation and sense the robot’s posture according to the change of the resistance value generated by the sensor deformation. The difference in the configured position of the actuator can result in a difference in the bending direction of the module, and the position of the sensor can be configured according to the actual application. The chamber position of the robot module and the bidirectional bending schematic are shown in Figure 2a–c. We define Figure 2b as forward bending and Figure 2c as backward bending. The length and size of the module can be adjusted appropriately, while the number of chambers at the top end and bottom end can be increased according to the configured needs of different robots. The well-designed module is manufactured by soft rubber with a 3D printer (WeNext Technology Co., Ltd., Shenzhen, China).

For soft animals without skeletons, muscles play an essential role in their locomotion. To simulate the good stretchability of their muscles, we used SCPAM strings to actuate the robots. The property of a SCPAM string made of twisted nylon sewing threads with a silver-plated surface can be briefly explained as follows [55,56,57]: When thermally stimulated, it will contract axially and expand radially, resulting in a significant contraction stroke; when the thermal stimulus is removed (cooling), the material will extend axially and shrink radially until it returns to its original state. In addition, the resistance of SCPAM string varies significantly during shrinkage or extension, so it is reasonable and feasible to select it as a sensor. The fabrications of 1-ply SCPAM string and 2-ply SCPAM string are shown in Figure S1 (see the Supplementary Material), along with their micrographs under the microscope.

### 2.2. Configuration of the Crawling Robot

**Figure 3:**
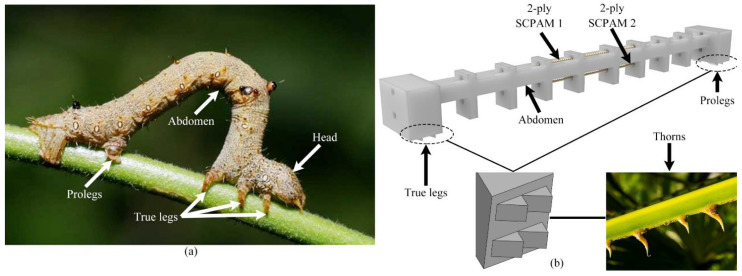
The inspiration for the design of the crawling robot. (**a**) A crawling inchworm and its tissues. (**b**) Soft crawling robot model and inspiration source of robot’s leg design.

In nature, inchworms can achieve continuous crawling locomotion in an unstructured environment. The main tissues or organs of the inchworm to achieve locomotion are the true legs at the front of its body, the abdomen in the middle, and the prolegs at the rear end. Figure 3a shows a real inchworm and its main tissues. The inchworm has only longitudinal and oblique muscle fibers in its abdomen and lacks circumferential muscles [58]. During locomotion, the longitudinal muscle fibers of the inchworm would contract, resulting in the bending and deformation of the trunk. Continuous crawling locomotion can be realized by exploiting the friction difference between true legs and prolegs with the ground. Inspired by this principle, we designed and configured a crawling robot using a single soft robot module. Figure 3b shows the rendered model of the soft robot. Two-ply SCPAM 2 is used as the longitudinal muscle to achieve bending and recovery of the robot, while another two-ply, SCPAM 1, serves as a sensor to sense the robot’s state.

From the microscopic to macroscopic scale, frictional anisotropy is ubiquitous in both biological and nonbiological systems. Such a feature can be easily found in plants and animals; for example, geckos use this structural feature to achieve agile climbing on ceilings, and the oriented distribution of wheat awns facilitates seed dispersal. The contacted surface of the structure is nonsymmetric and nonhomogeneous, and the friction force exhibits a large difference due to the difference in the friction direction [59,60]. Applying this anisotropic friction structure to the robot can reduce the complexity of the robot system. Inspired by the spine-like structure of thorns, we designed a set of 2 × 2 spine-like structures on the true legs and prolegs of the robot, respectively, which are arranged equidistantly and homogeneously along the radial centerline of the torso, as shown in Figure 3b. When the power is on, Joule heat is generated on the surface of the actuator, contributing to a contraction force that drives the true legs and the prolegs toward the middle, and a friction difference is generated in the spine-like structure on the underside of the legs due to anisotropic friction, resulting in anchoring of the true legs and forward locomotion of the prolegs. When the power is off, the actuator gradually returns to its original state, and the elastic energy stored in the soft robot body is released. The friction difference leads to an anchoring effect on the prolegs, and the true legs move forward [61]. The prototype of the crawling robot is shown in Figure S2 in the Supplementary Material.

### 2.3. Configuration of the Soft Gripper

**Figure 4:**
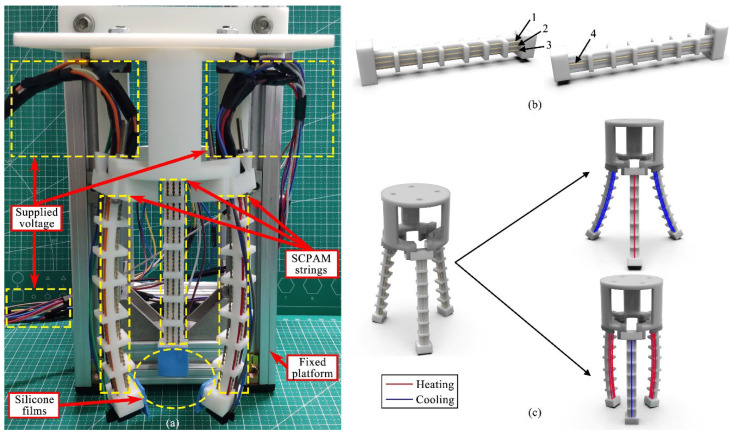
A prototype of soft robotic gripper, along with definitions of SCPAM string numbers and forward/backward bending of the gripper. (**a**) The soft gripper prototype fixed to the platform. (**b**) The definitions for numbering the SCPAM strings of each finger. The three SCPAM strings configured in the bottom end section are defined as numbers 1–3, and the SCPAM string configured in the top end section is defined as number 4. (**c**) Interpretation of the soft gripper’s bidirectional actuation capability. The top right image is defined as backward bending, while the bottom right image is defined as forward bending.

Similar to the crawling robot, the soft gripper is inspired by the tentacles of an octopus. Soft rubber is used to simulate the body, while SCPAM strings act as muscles to actuate the gripper. During the gripper’s configuration process, we found that low hardness of the finger would result in low stiffness, and a common limitation of SCPAM strings is that they often generate low force outputs [44]. The combination of these factors resulted in low effectiveness of the object grasping. Considering these factors, Shore hardness 90A robot modules are printed to configure the gripper fingers, while more actuators are added to output a larger force. The entire gripper consists of a base, three soft modules, and 12 SCPAM strings, while we cover the end of each finger with a layer of silicone film to increase the friction. The fingers are secured to a 3D-printed base by screws. A prestress of 0.6 N is applied to each SCPAM string at the bottom end of the module, while a prestress of 0.4 N is applied to SCPAM string at the top end section. The configured gripper prototype, as shown in Figure 4a, is fixed to the platform and then used for gripping tests. All the SCPAM strings configured in the module are defined and numbered in their spatial orders, as shown in Figure 4b. The three SCPAM strings configured in the bottom end of the module are defined as SCPAM 1–3, while the SCPAM string in the top is defined as SCPAM 4. The bidirectional actuation principle and sensing mechanism of the soft gripper are shown in Figure 4c. Similar to the forward and backward bending modes of the module defined in the previous section, there are two bending modes of the gripper. In the forward bending phase, SCPAM strings configured in the bottom of each module are used for actuation, while SCPAM strings configured in the top are not actuated. In the backward bending phase, SCPAM string 4 acts as an actuator, while SCPAM strings 1–3 are not activated. During the forward/backward bending phase, the position of the sensor can be configured according to the actual grasping applications. In addition to gripping objects with normal sizes, the presence of the backward bending mode allows the soft gripper to adapt to objects with greater sizes. Additionally, considering the long passive cooling time of the SCPAM string, activating the backward bending mode during the end of a gripping process allows the gripper to recover to its initial state quickly.

## 3. Experiments and Results

In this section, two prototypes of soft robots configured with soft robot modules are tested separately for their performances, and the effectiveness of the integrated actuation–sensing function is demonstrated. Before that, a basic characterization of SCPAM is conducted.

### 3.1. SCPAM Characterization

#### 3.1.1. Force Characterization

**Figure 5:**
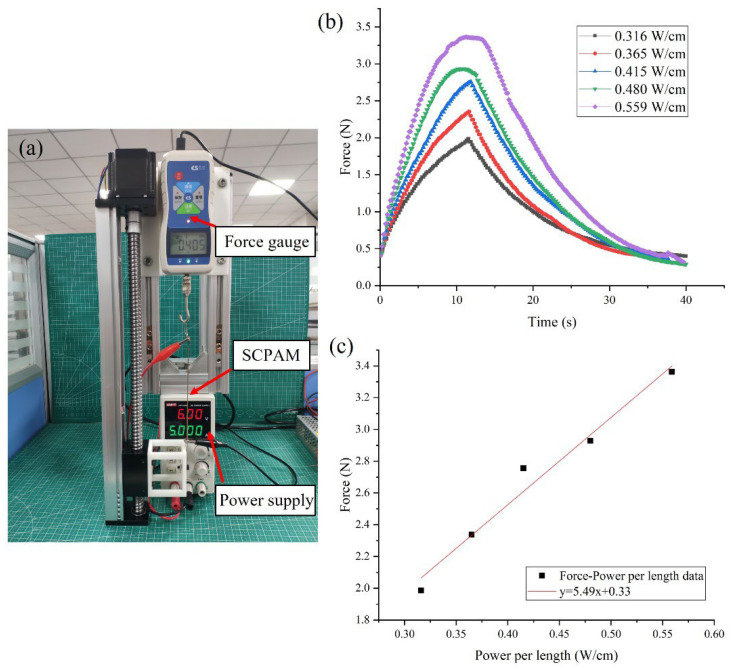
Force characterization of SCPAM. (**a**) Test platform. (**b**) Force–time curves under different power per length conditions. (**c**) Relationship between the maximum output force and power per length.

In this test, the output force of the two-ply SCPAM is investigated at different input powers. The power per length in this study refers to the electrical power applied to the SCPAM actuator normalized by its original unloaded length. The test platform is shown in Figure 5a. One end of the two-ply SCPAM is fixed to the screw linear guide, and the other end is connected to the probe of the force gauge (SH-III-20N, NSCING ES, Nanjing, China), while a pre-tension of 0.4 N is applied to SCPAM. SCPAM is supplied by a DC power supply (UTP1306S, UNI-T, Dongguan, China). The test power per length of SCPAM is 0.316 W/cm, 0.365 W/cm, 0.415 W/cm, 0.480 W/cm, and 0.559 W/cm, respectively. In this test, the SCPAM is powered on for 10 s and cooled off for 30 s at different input powers. The test results are shown in Figure 5a. Figure 5a shows that the output force is positively correlated with the unit input power, and the maximum output force is about 3.4 N at a unit input power of 0.559 W/cm. Figure 5c shows a linear fit of the maximum output force at different unit input powers. It should be noted that SCPAM generates a lot of heat quickly on its surface under high power input, and without a good thermal environment or temperature control, it may overheat and become damaged.

#### 3.1.2. Strain Characterization

**Figure 6:**
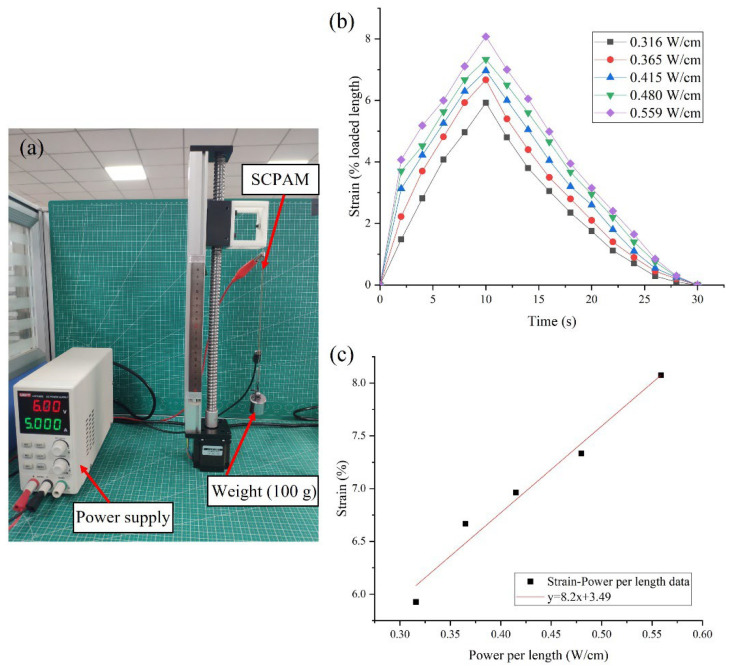
Strain characterization of the SCPAM. (**a**) Test platform. (**b**) Strain–time curves under different power per length conditions. (**c**) Relationship between the maximum strain and power per length.

To investigate the strain generated by the SCPAM at different input powers, the strain test platform is built as shown in Figure 6a. One end of the SCPAM is fixed to the screw linear guide, and the other end is connected to a 100-g weight to apply the preload. The initial length of the SCPAM in this test is 130 mm, and the loaded length is 135 mm. The strain is calculated by the ratio of the contracted length to the initial loaded length. The selection of the unit input power is consistent with Section 3.1.1. In the test, the SCPAM is heated for 10 s and cooled for 20 s. The test results are shown in Figure 6b. The maximum strain of the SCPAM is about 8% under a load of 100 g with a unit input power of 0.559 W/cm. Figure 6c shows a linear fit of the maximum strain for different input powers. It should be noted that the maximum strain of the SCPAM is influenced by various factors, such as the negative thermal expansion coefficient of the raw material used to fabricate the SCPAM, the spacing between coils due to the weight of the applied load during the coiling, heat treatment of the SCPAM, etc.

### 3.2. Locomotion Velocity Test of the Crawling Robot

Two control systems of the configured soft crawling robot with and without active cooling are built as shown in Figure S3. During the robot configuration and actuation test, we find that the actuator’s installation position has an impact on the actuation effect, so we conduct some further tests. The robot is segmented into a front end, rear end, and abdomen. In the following test, we explore the effect of the respective percentages on the robot locomotion by controlling the percentage of the front end, rear end, and abdomen length to the total length of the robot (the front end and rear end have same length).

Firstly, we define four modes:

- Mode A: Insert the actuator through eight guides, then fix the actuator’s ends at the first and eighth guides, respectively. The total length of the actuator is 111 mm.
- Mode B: Insert the actuator through the middle six guides, then fix the actuator’s ends at the second and seventh guides, respectively. The total length of the actuator is 81 mm.
- Mode C: Insert the actuator through the middle four guides, then fix the actuator’s ends at the third and sixth guides, respectively. The total length of the actuator is 51 mm.
- Mode D: Fix the actuator at the two middlemost guides, with a total length of 21 mm.

**Figure 7:**
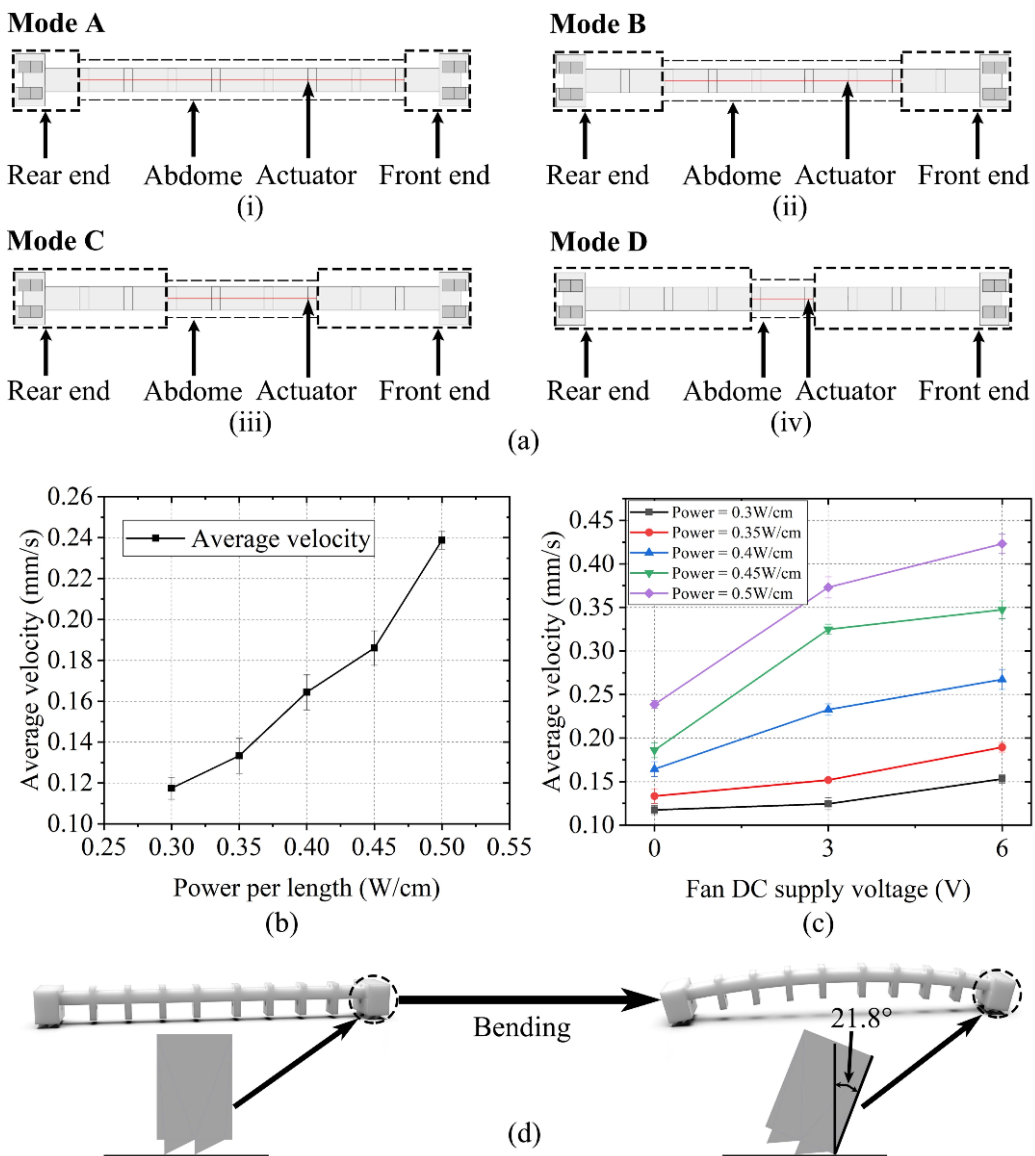
Locomotion velocity test of the crawling robot. (**a**) Four different percentage modes of the front end, rear end, and abdomen (the position marked with red line is the installed location of the actuator). (**b**) Locomotion velocity of the robot at different power inputs under mode C. (**c**) The relationship between the fan DC supply voltage and the robot locomotion rate for different input powers under mode C. (**d**) Threshold angle of the robot’s forefoot geometry to the ground is approximately 21.8°.

A schematic of the four modes is shown in Figure 7a.

The locomotion rate of the crawling robot under mode C at an indoor temperature (25 °C) is first tested. During the test, the input power is varied from 0.3 W/cm to 0.5 W/cm, with an interval of 0.05 W/cm. The actuator rapidly generates Joule heat to bend the robot when powered on, while passive cooling makes the robot take a long time to return to the initial state. Considering this, an actuation cycle is settled to 20 s, with a power on time of 5 s and power off time of 15 s. Five groups of experiments in which the power input varies from 0.3 W/cm to 0.5 W/cm are performed, and the robot moves for three cycles in each group. The locomotion rate of the robot increases with the increase of the power input. To avoid damage of the nylon thread by the excessive heat, the input power is upper-limited. The locomotion velocity of the robot at different input powers is shown in Figure 7b. The maximum crawling velocity of the robot at the maximum unit input power of 0.5 W/cm is about 0.24 mm/s. To decrease the recovery time for the actuator to its initial state, a cooling fan is added to shorten the cooling time. Since the added motor part is rigid, which will inevitably affect the flexibility of the crawling robot system, fan cooling is used as a simple contrast test only, and this method will provide a reference and comparison for us to develop a flexible cooling method in the future, although the crawling robot can work without active cooling. The relationship between the fan DC supply voltage and robot’s locomotion rate under different input powers applied to the robot is shown in Figure 7c. The crawling robot’s locomotion process is shown in Video S1.

The locomotion velocity tests of mode A, mode B, and mode D are performed in the same way. It is found that the maximum bending angle of the robot’s true legs has exceeded the threshold angle of the geometry to the ground surface (about 21.8°) when the same input power and power on/off frequency applied to mode A and mode B, respectively, as shown in Figure 7d. This deprives a geometry of function that provides frictional anisotropy, resulting in the inability of the robot to obtain stable locomotion along a certain direction. A test with the same input power and power on/off frequency applied to mode D is performed, and the result shows that the crawling robot moves extremely slowly under this mode. Therefore, these invalid locomotion modes (modes A/B/D) will not be discussed hereafter.

### 3.3. Locomotion Efficiency Analysis of the Crawling Robot

**Figure 8:**
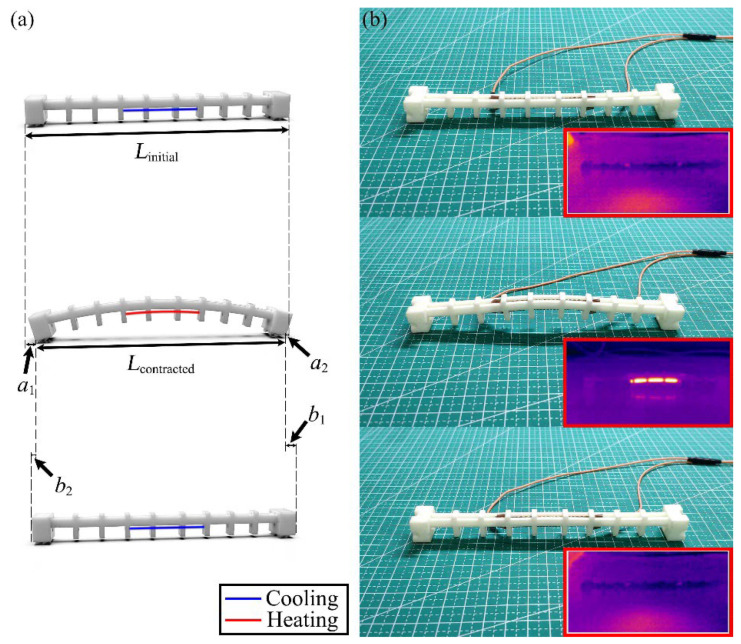
Locomotion efficiency analysis of the crawling robot. (**a**) Schematic diagram during one locomotion cycle of the crawling robot. The robot moves forward a short distance, accompanied by a slide. (**b**) The actual locomotion cycle and infrared thermograms during the locomotion of the crawling robot.

The special geometry configured at the bottom of robot’s ends allows the robot to achieve continuous crawling locomotion. The bending waves emitted by the actuator during the heating process are transmitted to the front end and rear end of the legs, leading to the variation of the angle between the geometry and the plane of the robot’s locomotion. The variation of the angle has an effect on the anisotropic friction property of the geometry. This results in a slide during the robot’s locomotion, with the front end moving slightly toward the rear end during the contraction process and the rear end moving a small distance backward during the extension process. Schematic diagrams of the whole locomotion cycle are shown in Figure 8a,b. Figure 8b also shows the infrared thermograms during the locomotion of the crawling robot.

Assuming that, when the robot completes the contraction process, the front end slides backward by *a*_2_ and the rear end slides forward by *a*_1_, when the robot completes the extension process, the front end slides forward by *b*_1_ and the rear end slides backward by *b*_2_. Ideally, *a*_2_ and *b*_2_ are both zero with the assumption that the robot moves without sliding. In this case, the ideal displacement obtained by the robot for each locomotion cycle is [61]:

*λ*_ideal_ = *L*_initial_ − *L*_contracted_, (1)

where *λ*_ideal_, *L*_initial_, and *L*_contracted_ represent the ideal displacement, initial length, and contracted length of the crawling robot, respectively.

However, the actual displacement obtained by the robot is smaller than the ideal displacement due to the unavoidable sliding during locomotion. The actual displacement *λ*_actual_ obtained by the robot for each locomotion cycle can be described as:

*λ*_actual_ = *a*_1_ – *b*_2_ = *b*_1_ – *a*_2_. (2)

Then, the linear locomotion efficiency *η* of the robot can be expressed as:

(3) $$\eta =\frac{\lambda_{\mathrm{actual}}}{\lambda_{\mathrm{ideal}}}\times 100\%.$$

The robot’s efficiency of linear locomotion under mode C can be calculated based on this formula. The linear locomotion efficiency of the crawling robot under mode C is 96%. As a comparison, the linear locomotion efficiency of the crawling robot under modes A/B at the same unit power input with an on/off frequency is 16.6% and 37.8%, respectively.

### 3.4. Sensing Test of the Crawling Robot

This section establishes a sensing model for the crawling robot to predict the crawling robot’s bending angle by measuring the resistance value of the sensor (SCPAM 2). During the stretching process of the SCPAM string, the change of the resistance value comes from the change of the SCPAM string’s length and cross-sectional area, as well as the change of the contact surfaces of two one-ply SCPAM strings used to configure the two-ply SCPAM string. During the thermal activation of the SCPAM string, the length and cross-sectional area of the SCPAM string also change, resulting in the change of its resistance value. However, it is difficult to use SCPAM string for sensing during its thermal actuation, because the temperature and resistance values of SCPAM string are tightly coupled. The temperature of the SCPAM string is highly nonuniform during thermal actuation. Other factors such as ambient temperature, air flow, and humidity can also affect the sensing performance during thermal actuation [44]. Considering the above factors, the stretching-induced sensing correlation at room temperature (25 °C) is established based on curve fitting instead of from a physical perspective.

**Figure 9:**
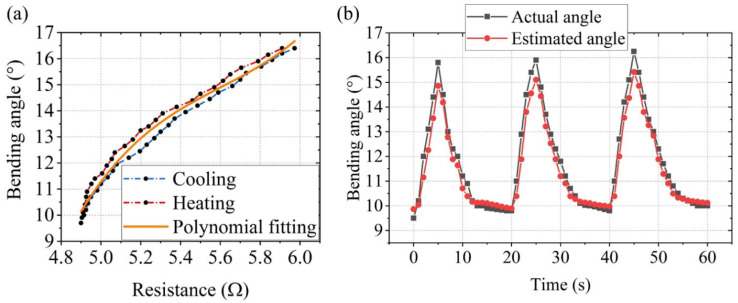
Sensing test of the crawling robot. (**a**) The third-order polynomial used to capture the relationship between the SCPAM string resistance and bending angle. (**b**) Graph of the sensing effect (estimated bending angles compared with actual bending angles).

Hysteresis nonlinearity appears in many materials, and it also exists in SCPAM strings [56,57,62]. This is due to the friction between the two one-ply SCPAM strings composing the two-ply SCPAM string, as well as between the coils of the single one-ply SCPAM string [44]. The corresponding bending angle—resistance value correlation is tested (three cycles of testing, heating for 5 s and cooling for 15 s). Schematic of the robot’s bending angle test is shown in Figure S4. A third-order polynomial is used to approximate the correlation between the bending angle and the resistance value to obtain a stretching-induced sensing correlation for the crawling robot. The corresponding bending angle–resistance value relationship is shown in Figure 9a, where the sensor resistance value increases with the increasing bending angle, and the curve demonstrates the hysteresis behavior of SCPAM string 1. The polynomial coefficients for the sensing experiment are given in Table 2.

It should be noted that the fitted third-order polynomial is performed at room temperature, and the effect of temperature is not taken into account in the actual prediction of the bending angle because: (1) The temperature of SCPAM 1 was highly heterogeneous under the effect of thermal radiation. If the contribution of thermal radiation to the resistance value of SCPAM 1 is included, a complex and dynamic model is required to predict the change of the resistance value of SCPAM 1, which will be a challenging task. (2) With a short power on time of only 5 s per cycle and a large, exposed area, SCPAM 2 dissipates heat well and is not much affected by thermal radiation, as evidenced by the infrared thermogram in Figure 8b.

We also performed a test to verify the sensing effect of the sensor. The procedure is as follows: during three locomotion cycles (at 0.5 W/cm unit power input), the resistance value of the robot is measured. Then, the bending angle of the robot is predicted according to the third-order polynomial, and finally, the estimated result is compared with the actual value. The specific result is shown in Figure 9b. In Figure 9b, we can see that error exists between the estimated angle and actual angle, which is originated from:

(1) Modeling error generated by the hysteresis behavior of the SCPAM string;
(2) Potential creep of resistance measurements over many cycles due to inherent nylon material properties and the estimation of the resistance value;
(3) Deviation of the camera position in the bending test;
(4) The static bending resistance relationship is used to predict the dynamic bending angle.

### 3.5. Output Force Test of the Finger

**Figure 10:**
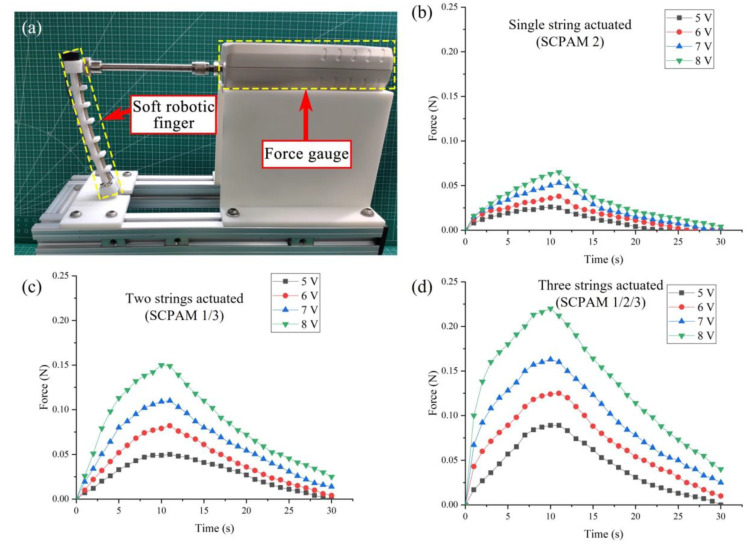
Output force test of the string. (**a**) The platform established to test the string’s output force. (**b**–**d**) The output force of the string’s end for time under different input voltages with the actuation of a single SCPAM string (SCPAM 2), two SCPAM strings (SCPAM 1 and 3), and three SCPAM strings (SCPAM 1–3), respectively.

To test the output force of a single finger, a force test platform is built, as shown in Figure 10a. A single finger and a force gauge (NSCING ES, SH-III-20N) are placed on a fixed platform, while the surface of the finger’s end is placed to contact with the force gauge’s fixture (no force is generated in the initial state). The finger output force tests are conducted separately with a single SCPAM string (SCPAM 2), two SCPAM strings (SCPAM 1 and 3), and three SCPAM strings (SCPAM 1–3). The result of the force test is shown in Figure 10b–d. Under the maximum input voltage of 8 V, the force output at the end of the finger is about 0.07 N (actuated by a single SCPAM string), 0.15 N (actuated by two SCPAM strings), and 0.22 N (actuated by three SCPAM strings). The definition of SCPAM numbers on a single finger can be found in the Section 2 of the paper.

### 3.6. Actuation–Sensing Integration Test of the Gripper

Actuation–sensing integration means that the soft gripper has sensing capability besides the conventional actuation capability. During the actuation–sensing integration test, SCPAM string 2 is always used as a sensor. The gripper uses SCPAM strings 1 and 3 as the actuators when the gripper bends forward. On the contrary, when the gripper bends in the backward direction, SCPAM string 4 functions as an actuator. Additionally, the bidirectional actuation capability of the gripper allows it to adapt to larger objects. Details on the definition of the forward/backward bending of the gripper can be found in Section 2.

**Figure 11:**
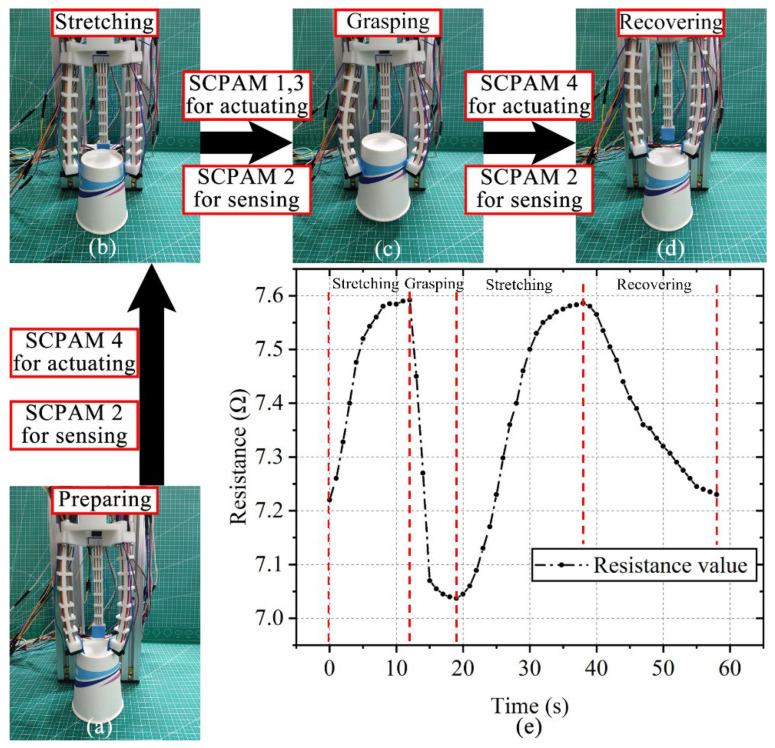
Actuation–sensing integration test of the gripper and resistance change of the sensor during the test. (**a**–**d**) The four stages of the gripper include the preparation stage, the stretching stage, the grasping stage, and the recovering stage, respectively. (**e**) The changes in the resistance value of the sensor during the test.

The control system to operate the soft gripper is shown in Figure S5. A paper cup is selected to test the gripper’s integrated actuation–sensing capability. As shown in Figure 11a–d, four phases of grasping process are defined, which are the preparing, stretching, grasping, and recovering stages. Among them, the gripper does not grasp an object during the preparing stage. During the stretching stage, the gripper bends backwards and approaches the object. At this time, SCPAM 4 at the top end of each finger is heated. The grasping stage indicates that the gripper bends forward and grasps the object, when SCPAM strings 1 and 3 act as the actuators. Similar to the stretching stage, SCPAM 4 is used as the actuator again at the final recovering stage. The sensor (SCPAM 2) is not actuated and continuously stretched throughout the gripping process, so its measurable resistance value changes can be used for strain sensing of the gripper without external sensors. Figure 11e shows the changes in the resistance value during the grasping process. At the stretching stage, the fingers are gradually stretched outward, and SCPAM 2 used for sensing is stretched due to the backward stretching, thus resulting in a continuous increase of its resistance. It is predicted that, when the angle of a finger continues to expand during backward bending, the resistance value of SCPAM 2 will increase continuously. However, since the prestress applied to SCPAM 4 is smaller than that applied to SCPAM 1–3, and there is only one actuator for backward bending, the bending angle of the gripper during backward bending is smaller than forward bending. The resistance value rises slightly and then stabilizes in a certain range. The maximum bending angle of the gripper during backward bending can approximately offset the effect of the initial bending angle due to the applied prestress in Figure 11b. At the grasping stage, the force generated by SCPAM 1 and 3 makes the finger bend inward, thus enabling the grasping of the paper cup. The sensor is compressed during this phase, and the resistance value gradually decreases until the finger touches the paper cup and then stabilizes. At the recovering stage, SCPAM 4 is used as an actuator again, allowing the gripper to quickly release the paper cup. The sensor is stretched again at this stage, and the resistance variation trend is the same as the stretching stage. The fingers are deformed to the maximum angle to ensure that the cup is released smoothly. Then, the actuators are powered off, and the fingers will return to their initial state under natural cooling, while the sensor resistance will also return to the value near the initial moment.

### 3.7. Gripping Test

**Figure 12:**
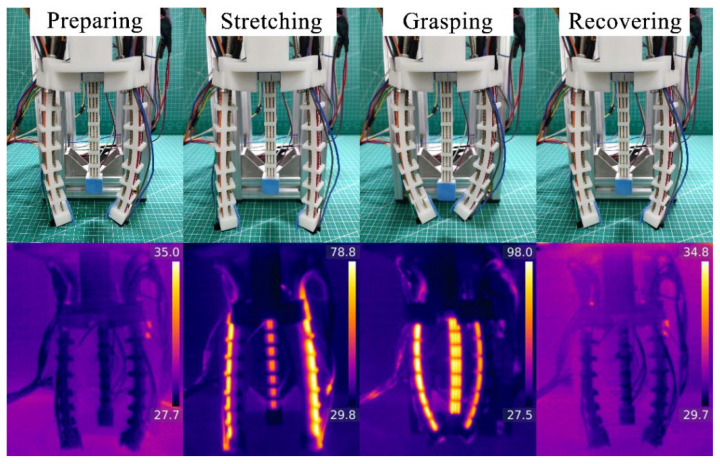
The whole gripping process without a load. Definitions of the four stages with their infrared thermograms during the grasping process.

**Figure 13:**
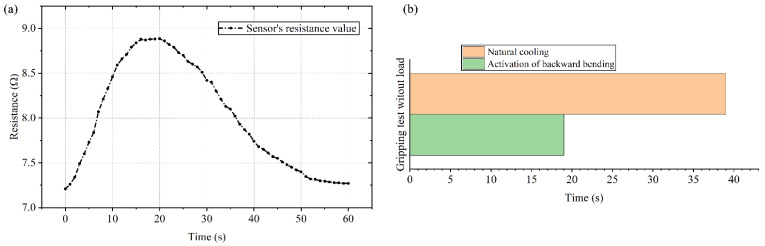
The changes in the resistance value of the gripper’s sensor without a load and recovery time comparison. (**a**) Resistance changes of the sensor during the grasping process (the stretching stage is not included). (**b**) The time required for the gripper to return to its initial state (natural cooling vs. activation of backward bending).

The whole gripping process and the infrared thermograms during the gripping process without a load are shown in Figure 12. Firstly, a sensing test of the gripper without a load is conducted with the voltage of 8 V. It should be noted that both the gripping test without a load and the following gripping object test do not include the stretching stage, and the SCPAM string 4 is always used as a strain sensor. Under the actuation of SCPAM strings 1–3, the fingers deform inward. The sensor’s deformation results in the change of its resistance value, as shown by the curve in Figure 13a. The resistance value gradually increases until the three fingers contact with each other at around 15 s. The fingers reach the limit bending angle at that moment, and the resistance value also remains stable when the finger’s bending angle stays constant. At 20 s, the power supply is turned off, and SCPAM strings 1–3 gradually recover to the initial state under passive cooling. At this time, the bending angle of the sensor also gradually decreases with the recovery of the finger, and the resistance value of the sensor also decreases. The whole cooling recovery time lasts about 40 s. In addition, the time required for the gripper to return to its initial state under the activation of backward bending during the cooling process is also tested. The result is shown in Figure 13b. Compared with passive cooling, backward bending allows the gripper to recover at a faster rate. The entire backward bending stage lasts about 20 s, which is half the time required for passive cooling.

**Figure 14:**
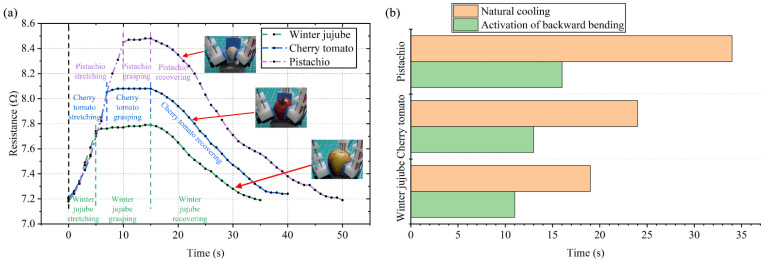
Resistance curves of the sensor based on different objects and their recovery time comparisons. (**a**) Changes in the resistance value during the gripping of pistachios, cherry tomatoes, and winter jujube, respectively. (**b**) The time required for the gripper to return to its initial state (natural cooling vs. activation of backward bending).

The capability of the gripper to grasp various objects is shown in Figure S6 in the Supplementary Material. With the proprioceptive sensing function, we can distinguish different objects based on the sensor resistance change during the gripping process of different objects. Three different objects are winter jujube, cherry tomatoes, and pistachios. The parameters of the different objects are shown in Table S1. The objects are directly grasped during the forward bending process of the gripper. The sensor resistance value variation curves during the grasping process are shown in Figure 14a, with the test voltage of 8 V and heating time of 15 s. When the object is gripped, the sensor’s resistance value increases rapidly during forward bending stage. Due to winter jujube’s comparatively larger size compared with cherry tomatoes and pistachios, a sensor’s resistance value becomes stable after rising up to a certain stage, and winter jujube contacts the finger at this stage. The relatively smaller size of pistachios makes the sensor’s resistance value continue to increase for a longer time. From the test results, the sensor resistance value variation curves are different due to the objects’ different sizes, and this provides us information to estimate the grasped object based on the sensor’s resistance value feedback. The grasping process of the gripper is shown in Video S2. Similar to in the gripping test without a load, the activation of backward bending during the passive cooling of the gripper can reduce the time required for the gripper to return to its initial state. As shown in Figure 14b, compared with natural cooling, backward bending improved the recovery velocity of the gripper with an increasing rate of 42.1% (for winter jujube), 45.8% (for cherry tomatoes), and 52.9% (for pistachios), respectively. This demonstrates the advantage of gripper’s bidirectional actuation capability in reducing the required recovery time.

## 4. Conclusions and Future Work

In this paper, a soft robot module with integrated actuation–sensing capability is proposed and intended for applications in a wide variety of soft robots. A crawling robot and a soft robotic gripper constructed from the soft robot modules are presented, whose performances are tested as well. The design of the crawling robot is inspired by inchworms and thorny plants in nature. The thorny structure at the bottom end endows the robot with the anisotropic friction mechanism required for crawling. The locomotion rate and efficiency of the crawling robot are tested. The relationship between the measured resistance value and the bending angle is approximated by a third-order polynomial. The established model can be applied for the sensing of the robot’s bending shape. The soft gripper consists of three same soft robot modules. The integrated actuation–sensing capability of the gripper is tested. Several different objects are tested to demonstrate the gripper’s grasping and sensing capabilities, and the results show distinct differences in the resistance–time curves generated by objects with different shapes and sizes.

In future study, the variable stiffness function will be added to the soft robot module so that the configured gripper can grasp a larger range of objects. In addition, theoretical modeling will be established to study the interaction between the two-ply SCPAM actuator and the soft body in a future study based on SCPAM modeling [63] and hyperelastic material modeling. The third-order polynomial model used to predict the bending angle of the crawling robot is not from a physical perspective, resulting in inaccurate predictions. Future targets are to make the stiffness of the module tunable within a range and to increase the output force of the module. The developed sensing model should consider various factors such as hysteresis, creep, resistance transient changes, etc. More soft robots constructed from the soft robot module will also be developed in our future research.

## Figures and Tables

**Table 1:** Summary of different actuation approaches for soft robot modules.

Reference	Actuation Method	Pros	Cons	Soft Robot Applications	Future Work (Envisioned Applications)
Huang et al. [22]	SMA	High power density, high reversibility, low operating voltage	Not soft material, hysteresis in phase transformation	Jumping, walking, rolling	--
Jin et al. [23]				Crawling, swimming, gripping	--
Robertson et al. [24]	Pneumatic	Light weight, soft, biocompatible	Needs bulky accessories, difficulty in controlling force	Gripping (suctioning), crawling, rolling, vertical climbing	--
Jiao et al. [25]				Gripping, manipulating, crawling, climbing, driving	--
Li et al. [26]	DEA	High elastic energy density, high strain, low Young’s modulus	High operating voltage	Rolling, creeping, crawling	--
He et al. [27]	LCE	Good mechanical properties, good flexibility, anisotropic behavior	Single stimulus response, hard to control deformation accurately, difficulty in remote control	Gripping, crawling	--
Minori et al. [28]				Lifting, crawling	--
Wang et al. [29]	SMP	High strain recovery, biocompatibility, biodegradability	Fatigue, high response time, low-medium power density	Gripping (three different grasping mechanisms)	--
Castledine et al. [16]	Motor & tendon	Easy to control, fast response velocity	Needs affiliated transmission mechanisms, adds rigid components	--	Manipulating, crawling
Tang et al. [30]	SCPAM	High power-to-weight ratio, inherent compliance, low-cost, small hysteresis. good customizability, long cycle life	Difficulty in controlling accurately, long cooling time, break at high temperature	Gripping	Swimming, crawling
Our work				Crawling, gripping	Manipulating, swimming

**Table 2:** Identified parameters of the third-order polynomials.

*p*_0_	*p*_1_	*p*_2_	*p*_3_
−1017.79	545.29	−96.66	5.75

## Data Availability

Not applicable.

## Supplementary Materials

The following supporting information can be downloaded at https://www.mdpi.com/article/10.3390/polym14112265/s1: Figure S1: The fabrications of 1-ply SCPAM string and 2-ply SCPAM string. Figure S2: Prototype of the crawling robot. Figure S3: Control systems for driving the crawling robot. Figure S4: Schematic diagram of the bending angle test. Figure S5: A control system for operating the soft gripper. Figure S6: The gripper grasping different objects. Table S1: Parameters of the different gripping objects. Video S1: The locomotion process of the crawling robot. Video S2: The grasping process of the soft robotic gripper.
