# Supplementary material for: A Proprioceptive Soft Robot Module Based on Supercoiled Polymer Artificial Muscle Strings
Source: Polymers (Basel). 2022 Jun 1;14(11):2265. doi: 10.3390/polym14112265 (PMC9182732; doi:10.3390/polym14112265)
Supplement: Supplementary file 1 [file polymers-14-02265-s001.zip › polymers-1726881 supplementary-final.pdf]

polymers

Supplementary Materials: A Proprioceptive Soft Robot Module Based on Supercoiled Polymer Artificial Muscle Strings

Yang Yang, Honghui Zhu [1], Jia Liu, Haojian Lu, Yi Ren and Michael Yu Wang

Fabrication of SCPAM

The manufacturing procedures of the SCPAM string used in this study are as follows (shown in Figure S1):

I. The two ends of the SCPAM string are knotted to connect a paper clip respectively. One paper clip is fixed to the motor, and the bottom end of the other paper clip is connected to a weight of 200 g. A small wooden stick attached to the bottom of the weight serves as a fixing device to prevent string's untwisting during the twisting process;

II. The motor is activated to twist the conductive twisted fibers to form 1-ply SCPAM string, and then the 1-ply SCPAM is folded along the middle to form 2-ply SCPAM string;

III. One end of the 2-ply SCPAM string is fixed to the motor and a weight of 400 g is attached to the other end. The string is trained by constantly powered on for 1 s and powered off for 9 s with a DC power supply (UNI-T, UTP1306S). A unit power of 0.5 W/cm is applied, which allows the 2-ply SCPAM string to obtain a better actuating effect.

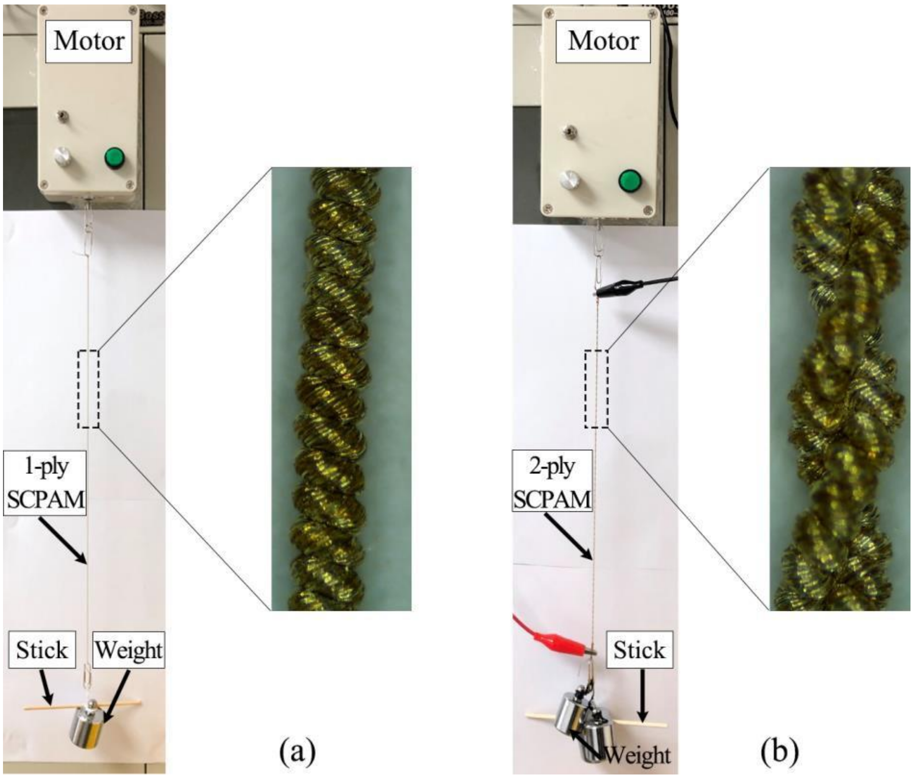

Figure S1. The fabrications of 1-ply SCPAM string and 2-ply SCPAM string. (**a**) Materials required for the fabrication of 1- ply SCPAM string and its micrograph under a microscope. (**b**) Materials required for the fabrication of 2-ply SCPAM stringand its micrograph.

Prototype of the crawling robot

In order to simulate good flexibility and bendability of the inchworm body, we 3D print a soft robot module with soft rubber (Shore Hardness 70A) as the crawling robot body, as shown in Figure S2a. The slot cut in the top of the main body is used toconfigure the sensor, and can also be utilized to observe the contraction of the sensor. The guides at the underside of the main body are designed to configure the actuator with a spacing of 12 mm between adjacent guides (except for the spacing of 15 mm between the middle two guides). The guides at the underside impart structural anisotropy to facilitate bending effect when the robot torso is stressed. Moreover, the guides can also reduce the local strain experienced during extreme bending to prevent elongation failure. The robot assembled by the main body, actuator and sensor adopts a modular design. This modular design is easier to assemble, less costly, easier to maintain. To obtain a large bending deformation, the actuator is embedded in the guide at the bottom, while the sensor is configured into the slot at the top. The actuator and sensor are fixed in the robot body via copper sleeves. To enable the robot to move normally, a pre-stress of 0.4 N is applied to the actuator. The components required for robot configuration and configured robot are shown in Figure S2b and Figure S2c, respectively.

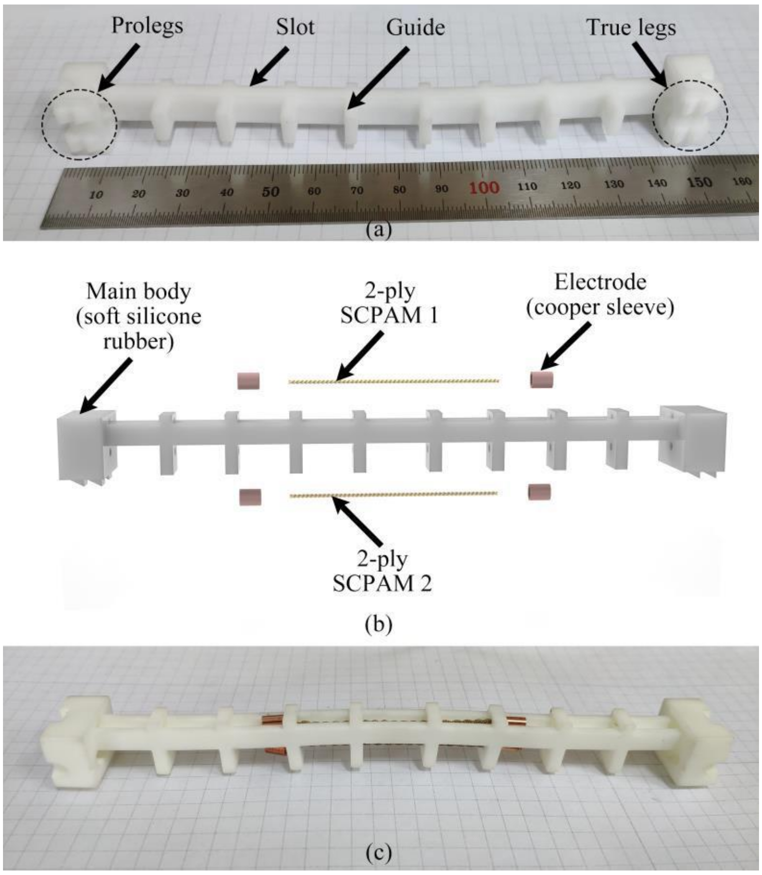

Figure S2. The configured crawling robot prototype. (**a**) Side view of the crawling robot's main body. (**b**) Componentsrequired to configure the robot. (**c**) Crawling robot after configuration.

Control system of the crawling robot

We build an open loop circuit for the crawling robot, which consists of an Arduino Uno and a motor driving module L298N. The PWM signals generated by the Arduino Uno are used as input signals to the L298N, which switch the external power supply that powers the robot actuator on and off. By programming, we can control the timing and duration of power-on and power-off to the actuator. During experiment, we find that SCPAM string is activated rapidly when powered on, which makes the crawling robot convert to the contracted state quickly. When power off, the actuator takes long time to fully recover to initial state due to the lack of active cooling, which has an impact on the robot's locomotion rate. Considering this factor, we add a DC motor to drive the fan in the open loop circuit to increase the locomotion rate of the robot. Two control systems of the crawling robot are shown in Figure S3.

For active cooling of the SCPAM actuator, A DC micro motor is used in this study with operating voltage of 1-6 V and speed of 17,000-18,000 r/min when 3 V voltage is applied as shown in Figure S3b. We find that during cooling period, the cooling time can be gradually decreased by increasing the fan speed, leading to a gradual decrease in the time required for one locomotion cycle of the robot, so it is crucial to control the time of active cooling. Too long active cooling time can increase the time required for one locomotion cycle, thus reducing the locomotion velocity. Too short active cooling time can reduce the time required for the robot's locomotion cycle, which results in a significant reduction in the robot's displacement generated by the next cycle due to the actuator not being sufficiently cooled, thus reducing the locomotion velocity. The crawling robot can get the optimal locomotion rate when the active cooling time is around 5 s according to tests. Besides, the robot's locomotion gait is soft and smooth, which differs from the rigid gait when no active cooling device is added.

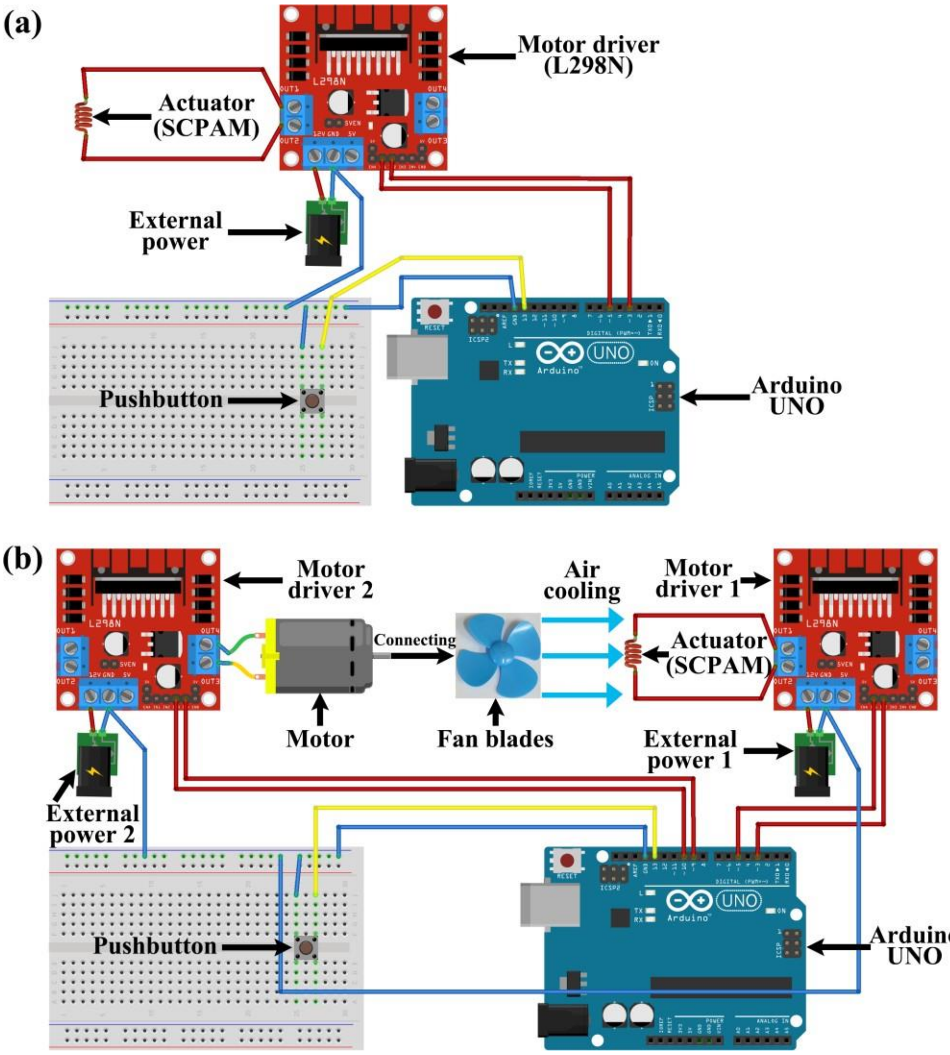

Figure S3. Control systems for driving the crawling robot. (**a**) The control system without the active cooling module. (**b**) The control system with active cooling module which adds a motor driving board and a DC motor. The DC motor is connected tothe fan blades to cool the SCPAM string.

Bending angle test set-up of the crawling robot

Schematic of the bending test is shown in Figure S4, where α indicates robot bending angle. Two ends of the actuator are connected to driving ports of motor driving board powered by an external DC power supply. At the same time, sensor ends are connected to a digital multimeter (UNI-T, UT890D+) for monitoring the change of resistance value. The whole locomotion process of crawling robot is recorded by a camera. In order to produce a relatively significant variation of the resistance value and make the robot generate a large deformation, we test the bending angle of the robot using unit power input of 1.2 W/cm (5s power on, 15 s power off).

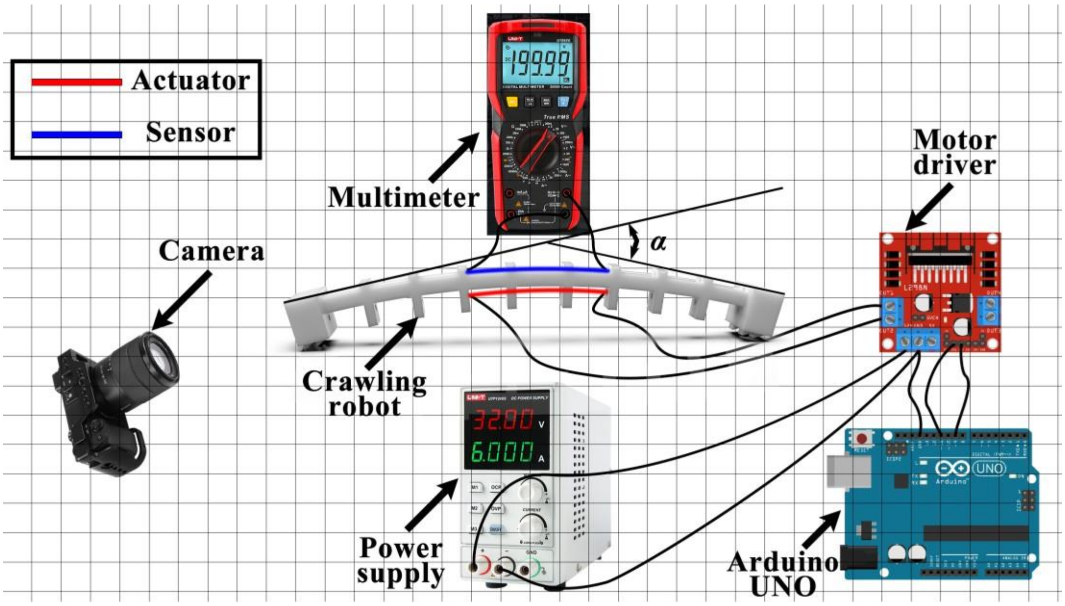

Figure S4. Schematic diagram of the bending angle test. A complete system is used to control the robot's locmotion and perform sensing test. The whole process is recorded by the camera.

Control system of the soft gripper

A complete system is built to control the operation of the soft gripper, as shown in Figure S5. Arduino (Arduino UNO R3) is used to communicate with PC, while high and low level signals generated by Arduino are utilized to control the opening and closing of relays. Two 8-relay modules (SONGLE, SRD-5VDC-SL-C) are powered by an independent power supply (Xiaomi, NDY-02-AN, DC 5.1 V/2.1 A). SCPAM strings are powered by a DC power supply (UNI, UTP1306S, DC POWER SUPPLY, 32 V /6 A) and each SCPAM string which can be controlled individually is connected to the NO terminal and COM terminal of the relay. The SCPAM strings obtain the corresponding heating and cooling effect under the relays' power-on/power-off switching mechanism, which allows the fingers of the gripper to obtain the bending and extending effect, thus ensuring completion of the gripping process.

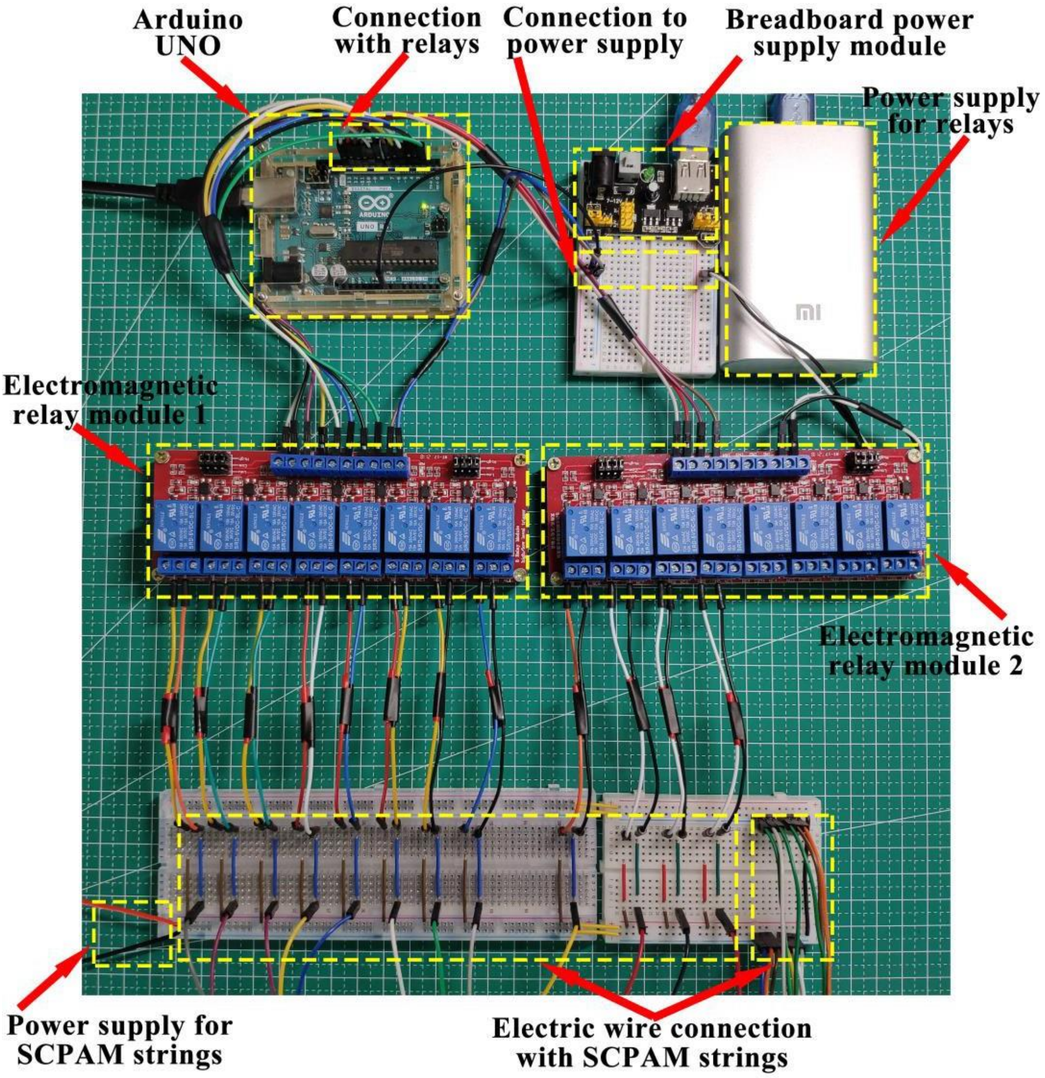

Figure S5. A control system for operating the soft gripper.

The gripper grasping different objects

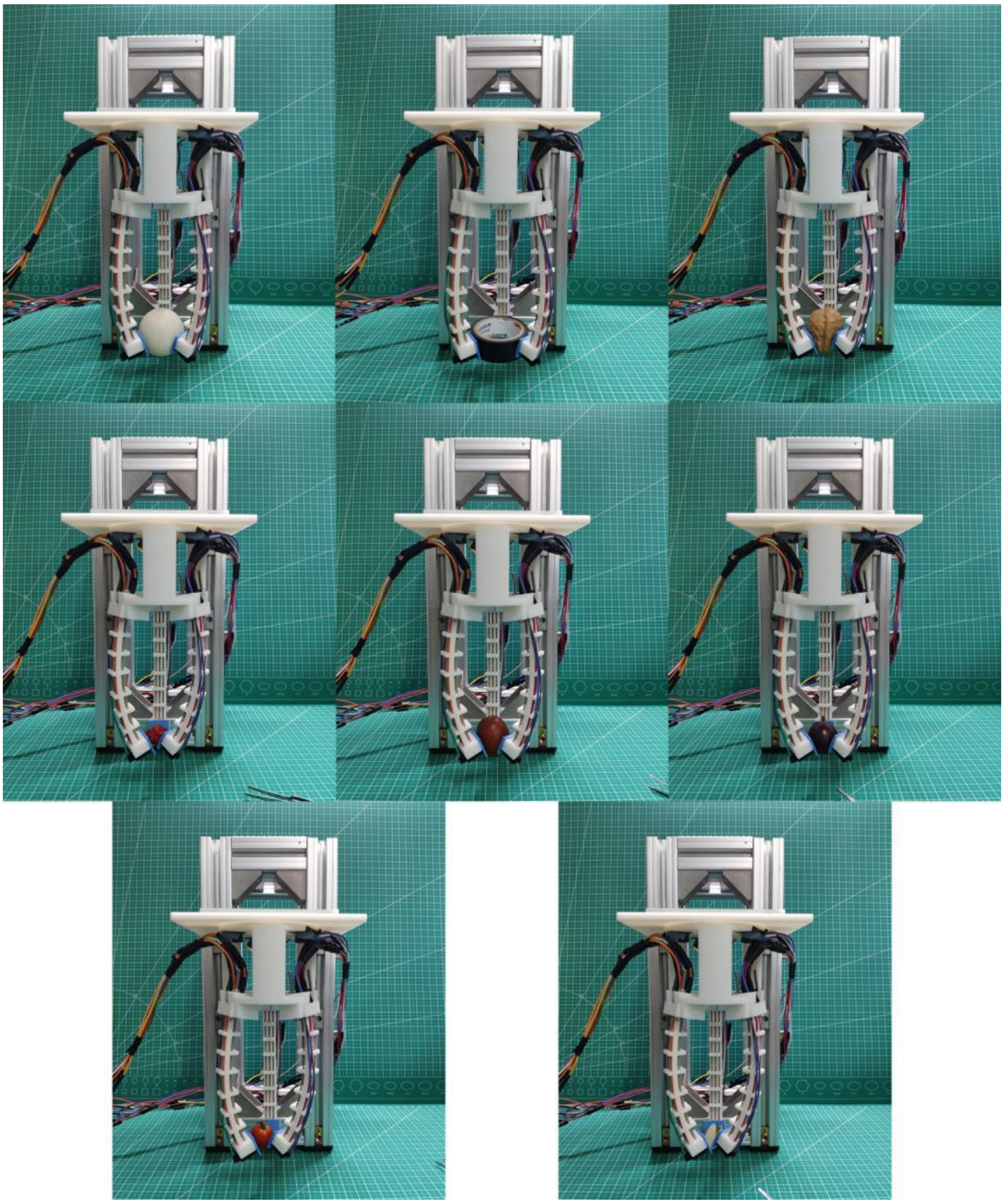

Figure S6. Grasping test of the gripper. Different grasping objects include ping pong ball, tape, walnut, Chinese date,winter jujube, grape, cherry tomato, and pistachio.

Table S1. Parameters of different gripping objects.

Object	Winter Jujube	Cherry Tomato	Pistachio
Quality	23 g	10 g	2.1 g
Maximum diameter in axial direction (long axis)	33 mm	25 mm	20 mm
Maximum diameter in radial direction (short axis)	28 mm	19 mm	14 mm
Time required to get in contact with the end of the gripper (8 V input)	4.8 s	7.1 s	9.7 s

Supplementary Video S1. The locomotion process of the crawling robot.

Supplementary Video S2. The grasping process of the soft robotic gripper.
